# Autotaxin in Pathophysiology and Pulmonary Fibrosis

**DOI:** 10.3389/fmed.2018.00180

**Published:** 2018-06-13

**Authors:** Ioanna Ninou, Christiana Magkrioti, Vassilis Aidinis

**Affiliations:** Division of Immunology, Alexander Fleming Biomedical Sciences Research Center, Athens, Greece

**Keywords:** autotaxin (ATX), lysophosphatidic acid (LPA), lysophosphatidic acid receptor (LPAR), g-proteins, pulmonary fibrosis

## Abstract

Lysophospholipid signaling is emerging as a druggable regulator of pathophysiological responses, and especially fibrosis, exemplified by the relative ongoing clinical trials in idiopathic pulmonary fibrosis (IPF) patients. In this review, we focus on ectonucleotide pyrophosphatase-phosphodiesterase 2 (ENPP2), or as more widely known Autotaxin (ATX), a secreted lysophospholipase D (lysoPLD) largely responsible for extracellular lysophosphatidic acid (LPA) production. In turn, LPA is a bioactive phospholipid autacoid, forming locally upon increased ATX levels and acting also locally through its receptors, likely guided by ATX's structural conformation and cell surface associations. Increased ATX activity levels have been detected in many inflammatory and fibroproliferative conditions, while genetic and pharmacologic studies have confirmed a pleiotropic participation of ATX/LPA in different processes and disorders. In pulmonary fibrosis, ATX levels rise in the broncheoalveolar fluid (BALF) and stimulate LPA production. LPA engagement of its receptors activate multiple G-protein mediated signal transduction pathways leading to different responses from pulmonary cells including the production of pro-inflammatory signals from stressed epithelial cells, the modulation of endothelial physiology, the activation of TGF signaling and the stimulation of fibroblast accumulation. Genetic or pharmacologic targeting of the ATX/LPA axis attenuated disease development in animal models, thus providing the proof of principle for therapeutic interventions.

## Introduction

**Figure 1:**
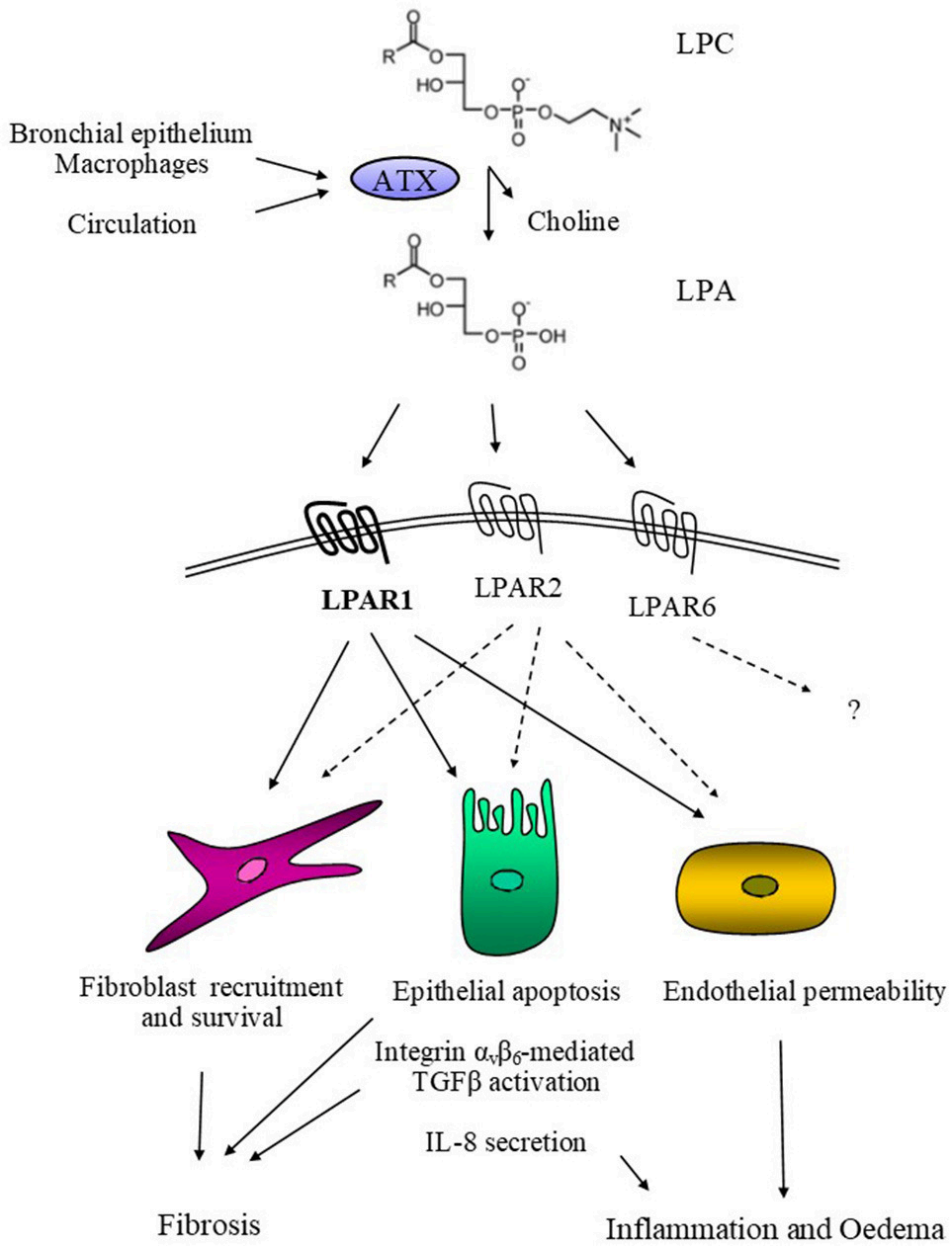
Schematic representation of ATX's mode of action in pulmonary fibrosis. ATX, derived from the bronchial epithelium and alveolar macrophages or extravasated from the circulation, catalyses the hydrolysis of LPC and the local production of LPA. In turn, LPA activates its cognate receptors LPAR1, possibly LPAR2, and hypothetically LPAR6, activating the corresponding G-protein-mediated signal transduction cascades. As a result, LPA induces epithelial apoptosis, the initiating pathogenetic event in modeled pulmonary fibrosis and possibly IPF. LPA also induces IL-8 secretion from epithelial cells, promoting inflammation, while it also stimulates endothelial permeability, thus promoting pulmonary oedema. Moreover, LPA stimulate the α_v_β_6_-mediated TGFβ activation leading to the activation and trans-differentiation of pulmonary fibroblasts, for which LPA is additionally a pro-survival and chemotactic factor.

ATX was first identified as an autocrine motility-stimulating factor, isolated from the supernatant of highly metastatic melanoma cells (1). Its cDNA cloning revealed that ATX was homologous to ectonucleotide pyrophosphatase-phosphodiesterase 1 (ENPP1), possessing phosphodiesterase activity *in vitro* (2); ATX was thus classified as ENPP2 in the ENPP (1–7) protein family, being the only secreted and not transmembrane member (3). In addition, several years later it was discovered that ATX is identical to the long elusive plasma lysoPLD (4, 5), and is now considered responsible for the synthesis of the majority of extracellular LPA (Figure 1).

## The *ENPP2*/*Enpp2* gene; expression and regulation

*ENPP2* consists of 27 exons and resides in the human chromosomal region 8q24 (6, 7), a region with frequent somatic copy number alterations in cancer patients, containing potential susceptibility loci for various types of cancers (8, 9). The 8q24 locus has been suggested to regulate the expression of the proto-oncogene *MYC*, also residing in the region (10). *In silico* analysis of publicly available genomic data at The Cancer Genome Atlas (11) indicated genetic alterations, mostly amplifications, of *ENPP2* in cancer patients, with the highest rates observed in ovarian (33%), breast (20%), liver (20%), and lung (11%) carcinomas (12). Moreover, a number of single nucleotide polymorphisms (SNPs) that associate with cancer susceptibility have been detected in or around *ENPP2* (9). Promoter regions of *ENPP2* were found hyper-methylated in primary invasive breast carcinomas (13), while inhibition of histone deacetylases 3 and 7 with trichostatin A also attenuated *ENPP2* expression in colon cancer cells (14), suggesting that *ENPP2* expression can be also amenable to epigenetic regulation. In mice, the highly (93%) homologous *Enpp2* gene is located in chromosome 15 and has a similar structure (15, 16).

A variety of cell types and/or tissues have been reported to express *ENPP2*/*Enpp2;* the highest mRNA levels in healthy conditions have been observed in adipose tissue, brain, and spinal cord, testis and ovary, followed by lung, kidney, and pancreas (15, 17–19), suggesting that ATX/LPA may participate in the homeostasis of these tissues. In disease states, increased mRNA expression has been reported in a large variety of cancer types and cell lines, as well as in different cell types in chronic inflammatory disorders (20).

Several transcription factors have been suggested to control *ENPP2*/*Enpp2* transcription in different cell types and pathophysiological states: Hoxa13 and Hoxd13 in mouse embryonic fibroblasts (21), v-jun in chick embryo fibroblasts (22), c-jun in soft tissue sarcomas (23), Stat3 in breast cancer cells (24), AP-1 in keratinocytes and neuroblastoma cells (25, 26), NFAT1 in melanoma and carcinoma cells (27, 28), as well as NF-kB in keratinocytes and hepatocytes (26, 29, 30). *Enpp2* mRNA stability has been reported to be controlled by the RNA-binding Proteins HuR and AUF1 (31), adding an extra level of regulation.

Several extracellular, mainly pro-inflammatory, factors have been suggested to stimulate *ENPP2/Enpp2* expression, many through the transcription factors indicated above: TNF in synovial fibroblasts, hepatocytes, hepatoma cell lines, and thyroid cancer cells (32–35), IL-1β in thyroid cancer cells (34), IL-6 in dermal fibroblasts (36), as well as galectin 3 in melanoma cells (27). Different TLR ligands, including LPS, CpG oligonucleotides and poly(I:C), were shown to stimulate *ENPP2* expression in THP-1 monocytic cells, likely involving an IFN autocrine-paracrine loop (37, 38). Lysophatidylcholine (LPC), a major component of cell membranes and oxidized lipoproteins as well as the enzymatic substrate of ATX, is a potent inducer of *Enpp2* expression in hepatocytes (32). On the other hand, the enzymatic product of ATX, LPA, as well as sphingosine 1 phosphate (S1P), have been suggested to create a negative feedback loop on *Enpp2* expression or activity, under certain conditions (34, 39).

## ATX isoforms, structure, and enzymatic activity

Alternative splicing of *ENPP2/Enpp2* exons 12 and 21 leads to five, all catalytically active, protein isoforms, named α to ε (15, 40). Isoform β is the most abundant one, likely accounting for the majority of ATX/LPA reported pathophysiological effects. Isoform δ is also abundant, lacking an exon 19 encoded tetrapeptide of unknown function, also missing in isoform ε. Isoform γ is brain specific, and contains an exon 21 encoded 25 aa insert of unknown function (20). Isoforms α and ε are much less abundant, while they contain a 52 aa polybasic insert, encoded by exon 12, that has been shown to bind to heparin and heparin sulfate proteoglycans (41). Proteolytic cleavage of a N-terminal hydrophobic sequence that functions as a signal peptide (42, 43) and glycosylation at asparagine residues (42–45), are necessary for secretion and optimal enzymatic activity.

ATX can be found catalytically active in most biological fluids, such as serum/plasma, bronchoalveolar lavage fluid (BALF), blister fluid, cerebrospinal fluid, synovial fluid, peritoneal fluid, and urine (20). The major source of serum ATX is likely the adipose tissue, as conditional genetic deletion of *Enpp2* in adipocytes resulted in a 38% decrease of plasma LPA (17), whereas ubiquitous heterozygous deletion results in a 50% reduction (46–48). Moreover, ATX has been shown to be secreted, in healthy conditions, from bronchial epithelial cells (49) and high endothelial venules (19), as well as choroid plexus epithelium cells (43), activated astrocytes and oligodendrocytes in the brain (50). Intriguingly, ATX has been also detected in exosomes (51), cell derived vesicles that have been suggested to mediate intercellular or cross-tissue signaling.

ATX consists of two N-terminal somatomedin B-like (SMB) domains, a central phosphodiesterase (PDE) domain and a nuclease-like domain (NUC) in its C-terminus (16, 52, 53). The SMB domains, stabilized by four pairs of disulphide bonds, likely mediate ATX binding to integrins, thus localizing LPA production to the cell surface (19, 52, 54–56). The PDE domain, which interacts with both SMB and NUC domains, contains the active catalytic site consisted of a threonine residue (Thr209/210, for mouse and human, respectively) and two zinc ions coordinated by conserved aspartate and histidine residues. It contains a hydrophobic lipid-binding pocket that can accommodate various LPC and LPA species and an open tunnel that could serve as an exit to LPA (53).

LPC, the enzymatic substrate of ATX, is highly abundant in the circulation, predominantly associated with albumin and lipoproteins (57). LPC is synthesized through the hydrolysis of phosphatidylcholine (PC) by phospholipases (PLA2, PLA1) and lecithin cholesterol acyltransferase (LCAT) enzymes (58). ATX has a preference for shorter and unsaturated fatty acid chains, depending on divalent cations such as Co^2+^ or Mn^2+^ (20, 53). Although ATX can also hydrolyze sphingosylphosphorylcholine (SPC, the precursor of S1P) and nucleotides *in vitro*, genetic and pharmacologic studies in mice established that the main enzymatic activity of ATX *in vivo* is LPC hydrolysis and the production of extracellular LPA (20, 53).

## LPA, receptors, and signaling

LPA consists of a glycerol backbone, a single fatty acyl chain of varying length and saturation, and a free phosphate group as a polar head. It can be found in most biological fluids, mostly following the expression profile of ATX (57, 59). LPA levels in serum are much higher than those in plasma, due to the release of LPC and other phospholipids from activated platelets during coagulation and their hydrolysis by ATX (60, 61). Moreover, the LPA concentration in plasma (~0.7 μM) is significantly lower than LPC's (~200 μM), while the predominant LPA species (18:2 > 20:4 > 18:1) are not analogous to the corresponding LPC ones (16:0 > 18:1/18:0 > 20:4); similar observations were made in BALFs (62). This can be likely explained by the slow release of LPA from ATX, due to its high affinity for LPA (39, 63), as well by the rapid turnover of LPA, as shown after the pharmacological inhibition of ATX *in vivo* (64, 65). Although there are other biosynthetic routes for LPA production, any increases in the extracellular LPA content of biological fluids and local sites can be attributed to the lysoPLD activity of ATX (58). On the other hand, a group of membrane-associated lipid-phosphate phosphatases (LPPs) have been suggested as negative regulators of LPA levels, adding an extra layer of regulation of its effects (66, 67).

LPA signals through at least six type I rhodopsin-like receptors (LPARs) that exhibit widespread, but differential, tissue distribution, as well as overlapping specificities (68). The orphan GPR87 and P2Y10 receptors (69, 70), as well as the receptor for advanced glycation end products (RAGE) (71) and the intracellular peroxisome proliferator-activated receptor γ (PPARγ) (72), have also been suggested to mediate LPA signaling. Little is known on LPARs functional conformation and possible associations; LPAR1 has been detected in lipid rafts (73, 74) and suggested to heterodimerize with CD14 (74) and CD97 (75).

LPARs couple with G-proteins, crucial molecular switches activating numerous signal transduction pathways (76). G-protein coupled receptors (GPCRs) is the largest family of cell-surface molecules involved in signal transduction, and their aberrant function has been linked with various human diseases, thus representing almost 50% of current therapeutic targets (77). Many *in vitro* studies, extensively reviewed elsewhere (20, 57, 61), have shown that LPA: stimulates the mitogenic Ras-Raf-MEK-ERK pathway and the PI3K pathway promoting cell survival through G_α*i*_; induces RhoA-mediated cytoskeletal remodeling, as well as cell migration and invasion through G_α12/13_ in cooperation with the G_α*i*_-mediated Rac activation pathway; activates phospholipase C, through G_α*q*_, with consequent production of second messengers. Of note, most *in vitro* effects of LPA were reported at concentrations much higher than the physiological concentrations, as found in healthy biological fluids, suggesting that they likely concern pathophysiological situations with increased levels of LPA. Overall, any LPA effect in each cell type will depend on its local concentration, regulated by ATX and LPPs, the levels of possible agonists and antagonists and the relative abundance of the different receptor subtypes.

## ATX/LPA in pathophysiology

Ubiquitous genetic deletion of ATX and abrogation of LPA production resulted to embryonic lethality in mice due to malformations of the vascular and neural systems (46–48, 78), indicating a major role for ATX in **development**; reviewed in Moolenaar et al. (79). Of note, the embryonic phenotype of ATX knock out mice did not resemble the phenotype of any of the individual LPA receptors knock out mice (68), suggesting that coordinated LPA signaling through various receptors is necessary for the observed ATX effects in embryonic development; non-catalytic effects of ATX in development are also possible especially in the neural system (50, 80). Accordingly, elevated ATX levels have been detected in human pregnancy, further modulated in pregnancy-related pathophysiological conditions (81–86).

Notwithstanding the necessity for ATX in embryonic life, induced genetic deletion or long-term pharmaceutical targeting of ATX in adult mice was shown to be well tolerated (18), indicating that the majority of ATX/LPA (>80%) is dispensable in **adult healthy life**. The remaining ATX-mediated LPA levels, together possibly with LPA produced via other routes (58) are likely adequate to maintain a healthy tissue homeostasis. Given the importance of ATX in embryonic development but not in adult life, the overexpression of ATX in a pathophysiological condition suggests ATX/LPA as a developmental pathway aberrantly re-expressed in pathophysiological situations.

One of the main features of the embryonic lethal phenotype of ATX knock out mice was the aberrant vascular system, as also seen upon ATX knockdown in zebrafish (87) and in line with the suggested role of LPA in **vascular homeostasis** (88, 89). A similar phenotype was also seen in the embryos of transgenic mice overexpressing ATX (90) and LPP3 knock out mice (91, 92) that sustain much higher levels of LPA than wt mice, suggesting that LPA levels should be tightly regulated during development. Of note, *G*_*a*13_^−/−^ embryos display similar impairments in the vasculature as the *Enpp2*^−/−^ embryos (46, 93), suggesting *G*_*a*13_ as the predominant G-protein mediating ATX/LPA effects in the vasculature. In adult life, LPA has been suggested to modulate endothelial cell physiology, through the stimulation of the expression of angiogenesis related genes and the modulation of their permeability (88, 89, 91). Beyond endothelial cells, LPA has a plethora of effects on other cells of the vessel wall, as well as on blood cells including platelets. Moreover, LPA is generated during mild oxidation of LDL, while its levels accumulate in atherosclerotic plaques, suggesting a role for ATX/LPA in **atherosclerosis** (94, 95).

The possible involvement of ATX/LPA in atherosclerosis is further underscored by the fact that the adipose tissue is a major source of systemic ATX, while its effects, through LPA, can classify ATX as an adipokine. Although the effects of ATX/LPA in adiposity are not clear (17, 96, 97), the ATX-LPA pathway has been suggested to participate in **obesity** related **insulin resistance** and the regulation of **glucose homeostasis** (98), with many implications for the pathogenesis of different metabolic disorders. However, the autocrine and/or paracrine effects of ATX/LPA in metabolism and the consequent effects in disease pathogenesis have not yet been fully explored.

ATX was first isolated due to its ability to promote the motility of melanoma cells (1). Accordingly, many xenograft studies have shown that ATX knock down in melanoma cells, as well as pharmacological inhibition of ATX and LPAR antagonism, attenuate the metastasis of melanoma cells in the lungs of mice, well establishing a role for ATX/LPA in **metastasis**; reviewed in Leblanc and Peyruchaud (99). Beyond melanomas, interaction of ATX with integrin α_v_β_3_ on tumor cells, has been reported to control the metastasis of breast cancer to the bone [reviewed in (56, 100)].

Transgenic over-expression of *Enpp2*, as well as *Lpar 1, 2*, or *3*, in the mammary gland resulted in spontaneous breast cancer development (101), indicating a role for the ATX/LPA axis in **breast cancer**. However, spontaneous carcinogenesis was only observed in aged mice, suggesting that ATX/LPA act synergistically with oncogenic age-related signals. Notwithstanding the conflicting reports on ATX levels in breast cancer, the source of ATX in breast cancer was suggested to be the adjacent mammary fat pads, rather than the cancer cells themselves (102), suggesting that ATX can have paracrine effects in cancer development. In the liver, genetic deletion of *Enpp2* from hepatocytes attenuated **hepatocellular carcinoma** (HCC) development, revealing ATX/LPA autocrine effects in hepatocyte metabolism (32, 103). Increased ATX expression has been reported in many other types of cancer, including thyroid and ovarian (20, 104).

Increased ATX levels have been also reported in neuroblastomas and glioblastomas (50) and given the abundant expression of the brain specific isoform ATXγ as well the neuronal defects of the *Enpp2*^−/−^ mice, a role for ATX/LPA in brain cancer seems likely, but it remains yet unexplored. However, another major role for ATX/LPA was revealed in the brain, as it was shown that PLA2/ATX-dependent LPA/LPAR1 signaling is crucial for the initiation of neuropathic pain (105, 106). Moreover, ATX was shown to modulate oligodendrocyte physiology and differentiation via catalytic and non-catalytic functions (50, 107). In this context, increased ATX and LPA levels have been reported in the sera and cerebrospinal fluid (CSF) of **multiple sclerosis** patients (108–110), while pharmacologic inhibition of ATX attenuated the development of experimental autoimmune encephalomyelitis (111).

Besides multiple sclerosis, ATX/LPA were shown to have a role in the pathogenesis of other chronic inflammatory diseases. Conditional genetic deletion of ATX from synovial fibroblasts or pharmacologic inhibition attenuated the development of inflammatory **arthritis** in animal models (33, 112), suggesting a major role for ATX/LPA in rheumatoid arthritis (113, 114). TNF-induced ATX secretion from synovial fibroblasts was shown to result in increased production of LPA which in turn stimulated, in an autocrine mode, cytoskeletal re-organization, proliferation, and migration of synovial fibroblasts (33), the main effector cells in disease pathogenesis. Moreover, increased ATX staining was noted in lymphoid aggregates, in line with the suggestion that ATX can be an adhesive substrate for homing lymphocytes, facilitating their transmigration across endothelial layers in different modes (19, 115–118). Further to the possible regulation of immune responses by ATX/LPA, LPA was recently shown to convert monocytes to macrophages (119).

Chronic inflammation of the liver, due to cytotoxic, viral or metabolic stimuli, was shown to stimulate ATX secretion from hepatocytes, while LPA was shown to activate hepatic stellate cells and to amplify pro-fibrotic signals (32). Conditional genetic deletion of *Enpp2* from hepatocytes or pharmacological inhibition of ATX, attenuated the development of fibrosis in a cytotoxic model (32). Increased ATX expression has been reported in patients with chronic liver diseases of different etiologies, suggesting ATX as a diagnostic marker of different forms of **liver fibrosis** (32, 120). ATX/LPA have been also implicated in the fibrosis of other tissues, such as renal fibrosis (121) and skin fibrosis (36, 122).

## ATX/LPA in pulmonary fibrosis

*Enpp2* has been suggested, using genome-wide linkage analysis coupled with expression profiling, as a candidate gene controlling lung function, development and remodeling (123). Accordingly, *Enpp2*^−/−^ mice were found to be embryonically lethal (46–48, 78), while *Lpar1*^−/−^ mice were shown to have reduced alveolar septal formation during development (124). In adult life, ATX is constitutively expressed by bronchial epithelial cells, in both humans, and mice, and can be detected in BALFs (49, 125). However, a 50% reduction of systemic ATX levels in the heterozygous *Enpp2*^+/−^ mice or genetic abrogation of bronchial *Enpp2* expression had no major phenotypic effect in the lungs of mice, suggesting that tissue homeostasis in health does not require large amounts of LPA (49, 126). On the other hand, transgenic overexpression of *Enpp2* from the bronchial epithelium or from the liver resulting to 200% increases of ATX systemic levels, had no gross phenotypic effect in the lung either, suggesting that ATX/LPA are not sufficient to induce lung damage *per se* (126).

Subsegmental allergic challenge of asthma patients induced ATX/LPA levels in their BALFs (127, 128), while pharmacologic inhibition of ATX resulted in a marked attenuation of Th2 cytokines and allergic lung inflammation in a triple-allergen mouse asthma model (128); conflicting reports have suggested both pro-inflammatory and anti-inflammatory roles for LPAR2 (128–130). Therefore, a role for ATX/LPA in asthma seems likely and consistent with early reports on LPA effects in the proliferation and contraction of airway smooth muscle cells (131, 132).

Increased ATX staining has been detected in lung tissue samples from IPF and fibrotic non-specific interstitial pneumonia (fNSIP) patients, compared to other interstitial diseases and especially control samples (49). ATX localized mainly within the hyperplastic bronchiolar epithelium, but it was also detected weakly on alveolar epithelium around fibroblastic foci, interstitial macrophages, and fibroblast like cells. On the contrary, ATX was minimally localized within both the inflammatory components of cellular NSIP lung samples and in areas of loose connective tissue, called Masson bodies, representing the pathogenic hallmark of cryptogenic organizing pneumonia. These two latter forms of idiopathic interstitial pneumonias have a propitious prognosis and an excellent treatment response to corticosteroids, indicating that ATX up-regulation is closely associated with more progressive and irreversible forms of pulmonary fibrosis, such as IPF/UIP and fNSIP (49). Of note, as ATX has been suggested to bind to integrins at the surface of platelets and cancer cells (52, 54, 56), it cannot be excluded that ATX can bind to the surface of lung cells via integrins, thus avoiding clearance while exerting locally-produced LPA effects. In turn, the levels of specific LPA species have been found moderately increased in BALFs and exhaled breath condensates collected from IPF patients (133, 134); however, larger studies are needed.

A similar ATX staining profile was observed in the lungs of mice treated with bleomycin (BLM) (49), the most widely used animal model of pulmonary inflammation and fibrosis (135, 136), while increased ATX levels were detected in the corresponding BALFs (49, 62). Conditional genetic deletion of *Enpp2* from bronchial epithelial cells (CC10^+^) and macrophages (LysM^+^), the main pulmonary cells expressing ATX, reduced BALF ATX levels and disease severity thus confirming the pulmonary ATX sources as well as establishing a pathogenic role for ATX. However, BALF ATX levels remained relatively high, while the modeled disease was not completely attenuated, suggesting additional, extrapulmonary sources of ATX. ATX levels in BALF correlated with total protein and albumin measurements, pointing to a possible extravasation of ATX from the circulation; paradoxically, no major effects in BLM-induced fibrosis development were noted in genetically modified mice with increased or decreased serum and systemic levels of ATX (49). Nevertheless, systemic pharmacologic inhibition of ATX, both with small molecules and DNA aptamers, decreased LPA levels, and attenuated pulmonary fibrosis (49, 137, 138). It should be noted that ATX inhibition with PAT-048 (Bristol Myers Squibb; WO2012024620) was reported to have no effects in BLM-induced pulmonary fibrosis (62), most likely due to experimental settings and compound characteristics. However, the therapeutic potential of targeting the ATX/LPA axis was recently re-evaluated, where yet another ATX inhibitor was shown to prevent BLM-induced pulmonary fibrosis (139). Many more small molecule ATX inhibitors have been reported (140, 141), however they are still not tested in animal models of pulmonary fibrosis. Intriguingly, the bile salt tauroursodeoxycholate (TUDCA) was recently reported to be a partial non-competitive inhibitor of ATX (142), suggesting that the previously reported therapeutic effects of TUDCA in BLM-induced fibrosis (143), could be due to ATX inhibition.

Moreover, an autocrine pathway linking ATX, LPA signaling and b-catenin was recently reported to contribute to fibrosis progression in lung allografts, one of the primary causes of long-term graft failure after organ transplantation (144). Pharmacologic ATX inhibition or LPAR1 antagonism decreased allograft fibrosis (144), further extending the therapeutic potential of targeting the ATX/LPA axis in lung fibroproliferative disorders.

In agreement with a pathogenic role of ATX/LPA in pulmonary fibrosis, ubiquitous genetic deletion of either *Lpar1* or *Lpar2* also abrogated BLM-induced disease development (133, 145). Pharmacologic antagonism of LPAR1 was shown to be beneficial for the treatment of BLM-treated mice (146), thus stimulating the respective on-going clinical trial (NCT 02068053). Moreover, simultaneous ATX inhibition and LPAR1 antagonism has been reported to have some additive effect in melanoma metastasis (147), warranting further investigation and/or optimization. Beyond LPAR1&2, LPAR6 is the highest expressing LPAR in the lung (not published data), but its possible role in pulmonary pathophysiology and fibrosis has not been explored yet (Figure 1).

Reduced numbers of TUNEL^+^ cells were noted in the alveolar and bronchial epithelium of BLM-treated *Lpar1*^−/−^ and *Lpar2*^−/−^ mice, suggesting that LPA, through LPAR1 and/or 2, promotes epithelial apoptosis (145, 148), the initiating pathogenetic event in this model (135) and, likely, in human patients (149). Interestingly, apoptosing epithelial cells post BLM were shown to express TNF that has a major contribution in the pathogenesis of the modeled disease (150), while TNF has been reported to stimulate ATX expression in other cell types (33, 35). Many other LPA possible effects in pulmonary epithelial cells *in vitro* have been reported and are detailed elsewhere (151), including the induction of IL-8 secretion resulting to neutrophil influx (152, 153).

LPA stimulation of normal human bronchial epithelial cells has been shown to increase stress fiber formation, and to reorganize integrin α_v_β_6_ at their ends leading to TGF-β activation (154). Integrin α_v_β_6_ has been shown previously to bind and activate TGF-β, a mechanism suggested to regulate pulmonary inflammation and fibrosis (155). TGF-β is the prototype pro-fibrotic factor with a well-documented involvement in the pathogenesis of both the human and the modeled disease, with effects on alveolar epithelial cell injury, myofibroblast differentiation, epithelial-to-mesenchymal transition, and ECM deposition and remodeling (156). TGF-β is produced by different cell types, including alveolar macrophages, while LPA was shown to induce TGF-β expression in pulmonary fibroblasts *in vitro* (145). Therefore, TGF-β activation and possibly expression is another important mechanism through which LPA promotes pulmonary fibrosis.

BALF isolated from BLM-treated mice stimulates the chemotaxis of pulmonary fibroblasts, which was found attenuated by more than 50% in the absence of *Lpar1* expression, indicating that LPA is a major fibroblast chemoattractant (133). The structural organization of LPAR2 has been suggested to govern gradient sensing and the directional migration of fibroblasts in response to LPA (157), while LPA-induced mTORC2-mediated PKC-δ phosphorylation was shown to be critically important for fibroblast migration and pulmonary fibrosis development (158). LPA has been reported to promote, through GPCR-mediated pathways, the cytoskeletal reorganization and proliferation of lung fibroblasts (151), mediated likely from LPAR2 (145) but not from LPAR1 (133). Moreover, LPA signaling, specifically through LPAR1, has been found to suppress, under certain conditions, the apoptosis of primary mouse lung fibroblasts induced by serum deprivation (148). Similar anti-apoptotic effects of LPA have been reported in many cell lines (151), further supporting a role for ATX/LPA in mediating pathologic fibroblast accumulation, the main pathogenetic event in IPF.

Calcium second messenger signals are essential for many critical cellular functions (159). In fibroblasts, calcium homeostasis and ionic mechanisms have been proposed to orchestrate many of their functions, including proliferation, secretion of extracellular matrix components, as well as TGF-β production and differentiation to myofibroblasts (160). In this context, transient receptor potential vanilloid 4 (TRPV4) Ca^2+^ channels have been shown to get activated in response to matrix stiffness, as found in fibrotic lungs (161), and to mediate fibroblast activation and differentiation (162). Interestingly, LPA is well known to stimulate Ca^2+^ influx and/or mobilization in many cells (163), while it was recently shown to directly activate a TRPV1 ion channel (164). Although, the activation was intracellular (164), transbilayer LPA movement has been suggested before in the activation of the nuclear PPARγ receptor (72). Therefore, LPA-induced alterations in calcium homeostasis can have dominant effects in the physiology of fibroblasts, as well as many other cell types.

One of the hallmarks of the observed protection from BLM-induced fibrosis in *Lpar1*^−/−^ mice was the attenuation of BLM-induced vascular leak, indicating a major role of LPA in promoting endothelial permeability upon damage (133). Accordingly, transgenic overexpression of ATX from the liver resulting to elevated circulating LPA levels induced a bleeding diathesis (55). However, the effects of LPA on endothelial permeability remain controversial, while different LPA receptors have been proposed to mediate different effects on endothelial physiology (23, 151, 164). Endothelial dysfunction mainly characterizes the development of atherosclerosis and cardiovascular diseases, however, interstitial lung diseases have all been reported to have a lung vascular disease component (165).

Therefore, ATX-mediated LPA production promotes pleiotropic effects in pulmonary cells stimulating the development of pulmonary fibrosis (Figure 1). Accordingly, ATX inhibition was shown to attenuate BLM-induced pulmonary fibrosis (49, 137, 138), thus providing the proof of principle for therapeutic interventions and stimulating the on-going clinical trial. In a phase 1 study, GLPG1690, a potent and orally bioavailable ATX inhibitor exhibiting a good PK/PD profile (137), was shown to be safe and well tolerated (166), as previously shown with another compound and genetic interventions in mice (18). An exploratory phase 2a study in IPF patients (FLORA; NCT 02738801) was just completed with promising results (expected to be published soon), leading to phase IIb, currently recruiting.

## Author contributions

IN and CM drafted the paper. VA edited it.

### Conflict of interest statement

The authors declare that the research was conducted in the absence of any commercial or financial relationships that could be construed as a potential conflict of interest.
